# Gut Feelings About α‐Synuclein in Gastrointestinal Biopsies: Biomarker in the Making?

**DOI:** 10.1002/mds.26480

**Published:** 2016-01-22

**Authors:** Claudio Ruffmann, Laura Parkkinen

**Affiliations:** ^1^ Oxford Parkinson's Disease CentreUniversity of OxfordOxfordUnited Kingdom; ^2^ Nuffield Department of Clinical NeurosciencesAcademic Unit of Neuropathology, University of OxfordOxfordUnited Kingdom

**Keywords:** biomarker, biopsy, enteric nervous system, Parkinson's disease, α‐synuclein

## Abstract

In recent years, several studies have investigated the potential of immunohistochemical detection of α‐synuclein in the gastrointestinal tract to diagnose Parkinson's disease (PD). Although methodological heterogeneity has hindered comparability between studies, it has become increasingly apparent that the high sensitivity and specificity reported in preliminary studies has not been sustained in subsequent large‐scale studies. What constitutes pathological α‐synuclein in the alimentary canal that could distinguish between PD patients and controls and how this can be reliably detected represent key outstanding questions in the field. In this review, we will comment on and compare the variable technical aspects from previous studies, and by highlighting some advantages and shortcomings we hope to delineate a standardized approach to facilitate the consensus criteria urgently needed in the field. Furthermore, we will describe alternative detection techniques to conventional immunohistochemistry that have recently emerged and may facilitate ease of interpretation and reliability of gastrointestinal α‐synuclein detection. Such techniques have the potential to detect the presence of pathological α‐synuclein and include the paraffin‐embedded tissue blot, the proximity ligation assay, the protein misfolding cyclic amplification technique, and the real‐time quaking‐induced conversion assay. Finally, we will review 2 nonsynonymous theories that have driven enteric α‐synuclein research, namely, (1) that α‐synuclein propagates in a prion‐like fashion from the peripheral nervous system to the brain via vagal connections and (2) that gastrointestinal α‐synuclein deposition may be used as a clinically useful biomarker in PD. © 2016 The Authors. Movement Disorders published by Wiley Periodicals, Inc. on behalf of International Parkinson and Movement Disorder Society.

Idiopathic Parkinson's disease (PD) is a neurodegenerative condition characterized by typical motor symptoms of tremor, bradykinesia, and rigidity, which enable its diagnosis in the majority of cases. Nonetheless, misdiagnosis can occur in up to 20% of cases, particularly in the early phases of disease.^1^ Thus, the postmortem detection by immunohistochemistry (IHC) of aggregation of α‐synuclein (ASN) in the brain coupled with neuronal loss in the substantia nigra remains the gold standard for the definite diagnosis of PD.

The progressive pathological changes, however, start years before the clinical onset of symptoms, and by the time of diagnosis, the disease is too advanced for any neuroprotective therapies to have full impact. In recent years, several studies have shown that ASN aggregation can also be detected outside the central nervous system (CNS), particularly in the enteric nervous system (ENS) of the gastrointestinal (GI) tract of PD patients.^2^, ^3^, ^4^, ^5^ The contrasting results and varying methodologies applied have recently been reviewed in detail by Visanji and colleagues.^6^ However, reflecting the fast development in this field, a number of studies on ENS ASN detection as a PD biomarker have since then added to the growing body of literature.^7^, ^8^, ^9^, ^10^ These have provided new evidence that GI ASN detection may facilitate a presymptomatic diagnosis of PD, with evidence of ASN accumulation in colonic biopsies up to 8 years prior to the development of motor symptoms,^7^, ^11^ which also suggests that ASN accumulation may start in the ENS long before it affects the brain. Recent studies have also supported the use of in vivo gastric detection of ASN to confirm a diagnosis of PD in clinically symptomatic subjects,^8^ whereas others have identified the appendiceal mucosa as a potentially useful source of tissue for detection of ASN.^9^ If confirmed, these findings could meet the current urgent need for a biomarker in PD, which could complement or perhaps even be superior to directly imaging changes in the nigrostriatal system to predict, confirm, track progression, and phenotypically stratify PD patients.^12^

However, a critical step in this process will be to develop a method that is sufficiently sensitive and specific. To date, both the use of different types of antibodies and morphological assessment of pathology have fallen short of accurately distinguishing between the physiological and pathological forms of the ASN protein. Given that ASN accumulation has repeatedly been detected in the GI tract of neurologically unimpaired individuals,^9^, ^13^, ^14^ the question of what constitutes the pathological species of ASN has increasingly emerged as the key outstanding question in the field and will be addressed throughout this review. For the purposes of clarity, we will use the term *ASN immunoreactive staining* (ASN‐IRS) to describe any kind of ASN‐positive staining described in the literature (ie, aggregates, inclusions, fibers, etc.). Based on the morphology of staining, we will subdivide this into somatic (ie, round, Lewy body–like inclusions or diffuse staining in the perikaryon of ganglionic cells), neuritic (ie, thread‐like staining in dendrites and axons), or synaptic (ie, dot‐like staining in the neuropil).

## Sensitivity and Specificity of Enteric Neuronal ASN‐IRS in PD

Although initial studies with small cohorts showed promisingly high sensitivity and specificity rates,^3^, ^5^ this was not sustained in subsequent large‐scale studies (Table 1; also see review by Visanji et al).^7^, ^15^ Sensitivity as low as 10% was reported in the largest cohort to date, in which ASN‐IRS was detected in biopsies from various levels of the GI tract in only 7 of 62 PD patients.^7^ However, case selection was potentially less rigorous in this study, which relied entirely on retrospective review of medical records, and analyzed tissue was derived from biopsies done for reasons unrelated to PD. On the other hand, studies examining “ad hoc” fresh biopsies from patients included after clinical examination by a neurologist collectively obtained a sensitivity of 86%.^2^, ^3^, ^5^, ^8^, ^14^ There is also significant discrepancy in specificity, with some studies reporting an optimal 100% specificity,^2^, ^3^, ^4^, ^5^, ^7^ whereas others detected ASN‐IRS in more than 50%^15^ or as many as 82%^14^ of control subjects. Visanji and colleagues found 9 of 11 control individuals to have ASN‐IRS in both colonic and rectal biopsies, which could not be distinguished from PD patients by any specific feature such as location, area, or intensity.^14^

**Table 1:** Previous studies on GI ASN pathology

Reference	Antibody	Technique	Tissue source	Organ‐tissue (region)	Staining	
					PD^a^	Non‐PD^b^
2006 Braak	Syn‐1	IHC	Postmortem	Stomach (fundus, cardia, corpus)	5/5	HC 0/5
2006 Bloch	LB509	IHC	Postmortem	Sacral plexus, vagus, paravertebral ganglia, esophagus	—	ILBD17/98
2008 Lebouvier	P129Syn	DIF	Ad hoc fresh biopsy	Colonic submucosa (ascending)	4/5	HC 0/5 Chronic constipation 0/3
2009 Beach	P129Syn	IHC	Postmortem	Multiorgan (including GI tract)	11/17	HC 0/23 DLB 5/9; ADLB 1/19; ILBD 1/7
2010 Lebouvier	P129Syn	DIF	Ad hoc fresh biopsy	Colonic submucosa (ascending and descending)	21/29	HC 0/10
2012 Pouclet	P129Syn	DIF	Ad hoc fresh biopsy	Colonic submucosa (ascending and descending) + rectum	17/26	HC 0/9
2012 Shannon	LB509	IHC DIF	Archival biopsy material	Colonic submucosa	3/3	HC 0/23
2012 Shannon	LB509	IHC	Ad hoc fresh biopsy + archival	Colonic submucosa (sigmoid)	9/9	HC 2/24 Irritable Bowel 3/24
2012 Bottner	P129Syn	IHC DIF	Ad hoc fresh biopsy	Colonic submucosa (sigmoid) + rectum	‐	Colorectal carcinoma or prolapse 11/11
2013 Gold	KM51	IHC	Postmortem	Colonic submucosa	10/10	HC 40/77 AD 3/8
2014 Hilton	KM51	IHC DIF	Archival biopsy material	Various GI (esophagus, stomach, duodenum, colon)	7/62	HC 0/161
2014 Sanchez‐Ferro	KM51	IHC DIF	Ad hoc fresh biopsy	Stomach (antrum, pylorus)	17/20	HC 2/23
2014 Gelpi	KM51	IHC	Postmortem	Multiorgan (including GI tract)	8/10	5/5 DLB 0/8 AD
2014 Gray	LB509	IHC DIF	Surgical specimens	Stomach, colon	—	HC 20/20
2015 Visanji	LB509 P129Syn	IHC PET blot	Ad hoc fresh biopsy	Colonic submucosa (descending) + rectum	22/22	HC 9/11

a ASN‐positive staining in PD cases.

b ASN‐positive staining in non‐PD subjects.

Abbreviations: AD, Alzheimer's disease; ADLB, Alzheimer's disease with Lewy bodies; DIF, double immunofluorescence; DLB, dementia with Lewy bodies; HC, healthy controls; IHC, immunohistochemistry; ILBD: incidental Lewy body disease; P‐ASN‐Ab, antibody reactive for phosphorylated ASN; PET blot, paraffin‐embedded tissue blot; T‐ASN‐Ab, antibody reactive for total ASN.

To our knowledge, two studies have reported in vivo ASN‐IRS exclusively in neurologically unimpaired subjects.^9^, ^13^ In the most recent of these, Gray and colleagues reported somatic, diffuse ASN‐IRS in the appendices of 20 of 20 neurologically unimpaired subjects but also abundant neuritic and synaptic ASN‐IRS in the lamina propria with an apicobasal gradient of increasing fiber density.^9^ It is unlikely that prodromal PD underlies the ASN‐IRS in all neurologically unimpaired subjects described in these studies; thus, these findings raise concerns about the use of colonic biopsies to diagnose PD.

## Biopsy Sampling

**Figure 1:**
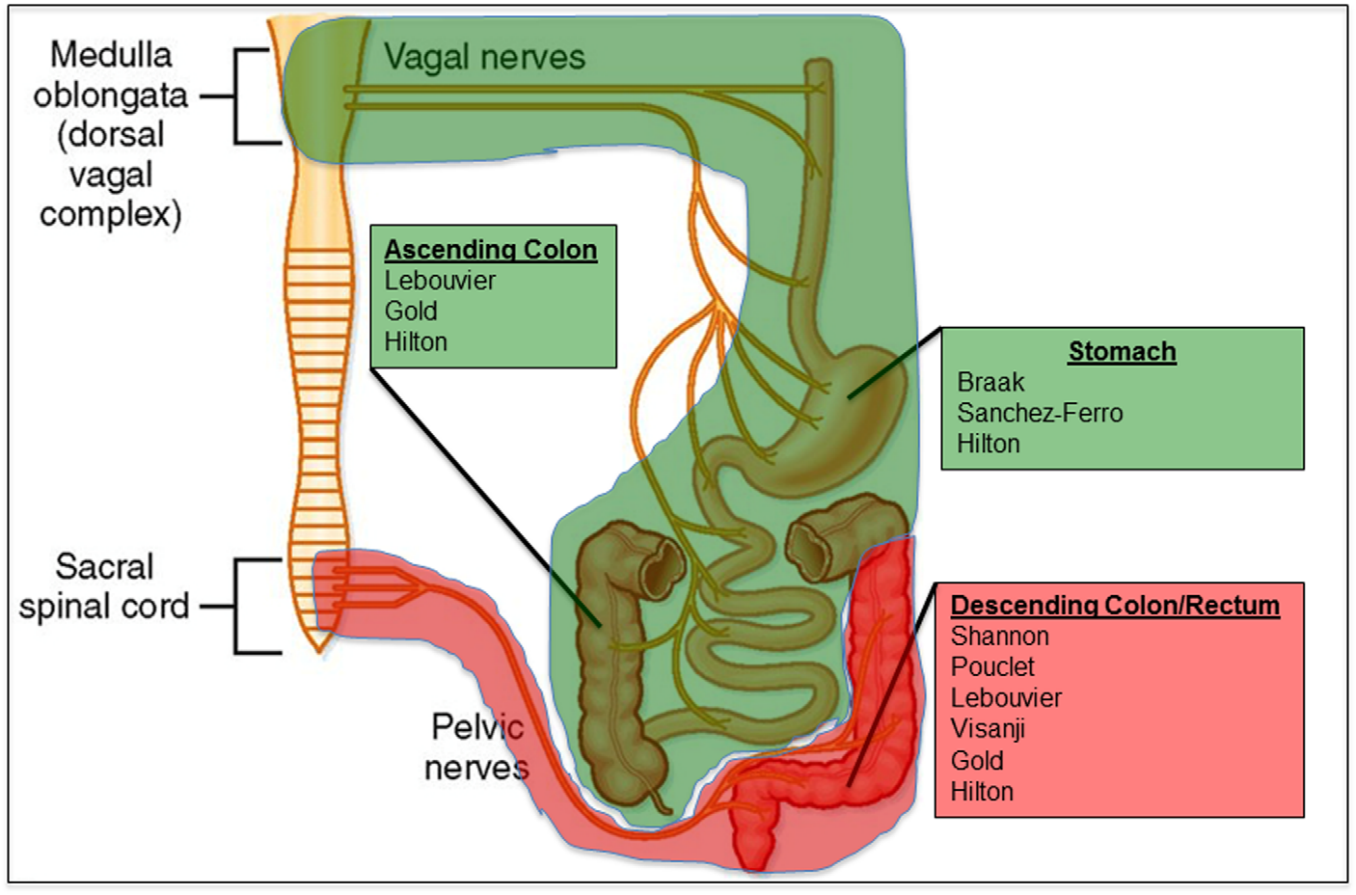
Previous studies and GI parasympathetic innervation. Parasympathetic innervation of the GI regions shaded in green originated in the brain stem nuclei of the vagus nerve, whereas parasympathetic innervation of the GI tract shaded in red was provided by nerve fibers originating in the sacral plexus. Previous studies have often investigated regions innervated exclusively by the sacral plexus (listed below each region). Modified with kind permission from BM Koeppen. Berne and Levy's Physiology, 6th edition (Mosby/Elsevier; 2010).^75^ [Color figure can be viewed in the online issue, which is available at wileyonlinelibrary.com.]

One highly variable parameter between studies is the site of the GI tract biopsy (Fig. 1). Several different regions of the GI tract have been harvested including submandibular glands,^16^ stomach,^8^ appendix,^9^ ascending and descending colon,^3^ sigmoid colon, and rectum.^5^, ^14^ Both postmortem^17^, ^18^, ^19^ and in vivo^3^, ^4^ studies have reported a higher density of ASN‐IRS in the rostral regions of the GI tract compared with the more distal ones. However, findings from more recent studies would seem to question this hypothesis. Hilton and colleagues reported the absence of ASN‐IRS in the esophagus, whereas 8% of biopsies from the stomach and 13% of both small and large intestine showed ASN‐IR staining,^7^ suggesting a caudo‐rostral gradient, if any. Furthermore, Visanji and colleagues found equally prevalent ASN‐IRS when comparing biopsies taken 5 cm (rectum) and 20 cm (sigmoid colon) from the anal verge.^14^ Finally, Gray and colleagues showed that neuritic ASN‐IRS was much more abundant in the mucosa of the appendix than in stomach, ileum, or colon, highlighting appendix as the optimal anatomical locus.^9^

Several reports have also demonstrated the impact of the amount of tissue analyzed on the likelihood of capturing ASN‐IRS.^8^, ^18^, ^19^ Of note, routine 4‐ to 6‐μm‐thick paraffin sections of the GI biopsies do not enable visualization of neuritic ASN‐IRS such as those seen on 150‐μm‐thick cryosections.^18^ Most recently, in a study examining gastric biopsies Sanchez‐Ferro and coworkers reported a minimum cutoff of 4 slides per case to reliably find ASN‐IRS in PD patients.^8^ Thus, increasing the number of consecutive sections analyzed could considerably facilitate depicting the scattered ASN‐IRS. These considerations should be borne in mind when interpreting reported results. For example, the 100% specificity reported by Hilton and coworkers could be at least partly due to the assessment of a single section for each control case as opposed to 2 sections for each PD case.^7^ Conversely, access to larger amounts of tissue, including the deeper myenteric plexus in the postmortem studies, may partly underlie the higher incidence of ASN‐IRS in neurologically unimpaired individuals reported by Gold and colleagues.^15^

To date, studies have mostly used qualitative assessment of sections to define ASN‐IRS, and only 2 studies developed semiquantitative grading scales to measure the severity of staining (also see the review by Visanji et al).^3^, ^15^ Virtual microscopy and whole‐slide imaging could allow a more robust quantitative analysis of enteric ASN‐IRS and potentially facilitate definition of reliable cutoff scores to distinguish PD patients from controls. To date, 1 study has used computerized scanning of stained sections, but the authors did not attempt software‐based quantification of the density of ASN‐IRS on single sections.^14^ Such software‐based image analysis could also allow adjustment for significant variability between biopsies in surface area, density of nervous tissue, and representation of different organ layers (mucosa, submucosa, muscle, etc.), all of which may affect the yield of ASN‐IRS.

## Localization of ASN‐IRS to Neurons in the ENS

In addition to neurons, ASN is physiologically expressed in hematopoietic,^20^ endothelial, and neuroendocrine cells^21^ and in muscle fibers.^22^ Therefore, the detection of nonneuronal ASN does not necessarily indicate a disease state. Conversely, we cannot currently rule out the potential pathogenic role of nonneuronal ASN either. Indeed, neuroendocrine, smooth muscle, and plasma cells, which are highly represented in the GI tract (as opposed to the CNS) and located in close proximity to neuronal elements, could represent possible sources of ASN. ASN may be taken up by neurons from neighboring cells or from the extracellular space, and further studies assessing transport of ASN between neuronal and nonneuronal cells would be informative.^23^, ^24^, ^25^

Interestingly, 2 recent studies excluded a significant proportion of cases from analysis because of arbitrarily determined subthreshold staining with neuronal markers.^7^, ^8^ We would argue that neuronal staining should provide data on density of nervous tissue, but that it should not guide inclusion criteria for staining with ASN‐reactive antibodies (Abs).

In the context of neuronal ASN, it is important to note that although some Lewy bodies (LBs; ie, perikaryal ASN aggregates) have been reported in the ganglionic cells of enteric plexuses,^7^, ^17^ they are rare, and ganglionic neurons prevalently show variably intense, diffuse, or punctate staining with irregularly distributed clusters of rings.^15^ Furthermore, endoscopic biopsies are willingly kept superficial for safety reasons (ie, to prevent bleeding). Consequently, they usually only contain mucosa with possible inclusion of muscularis mucosa and the submucosal layer (Meissner's plexus). Thus, in these biopsies most ASN aggregation is seen in the peripheral nerve endings of the lamina propria, which ramify along the enteric glandular structures. This morphology of staining can be challenging to distinguish from the above‐mentioned nonneuronal profiles,^26^ unless a proper neuronal marker is used.

Previous studies have used a variety of neuronal markers alone or in double immunofluorescence (DIF) with ASN‐Abs. Lebouvier and colleagues used an antibody reactive for the heavy chain of neurofilaments,^3^ whereas Shannon and coworkers used an antibody reactive for substance P, a generally accepted marker of postganglionic excitatory neurons in the colonic submucosa, and cuprolinic blue dye (quinolinic pthalocyanine), a staining technique that detects enteric neuronal soma and nuclei but is less specific for the fibers.^5^, ^11^ Anti‐Hu C/D and anti‐PGP 9.5 Abs have also been used, as they are considered panneuronal markers,^13^ whereas others have used Abs reactive for the S100 protein, which is actually reactive to glial cells.^7^, ^8^ Finally, several studies did not use any neuronal marker and relied exclusively on morphology to identify neurons and nerve cell processes.^14^, ^15^, ^18^

**Figure 2:**
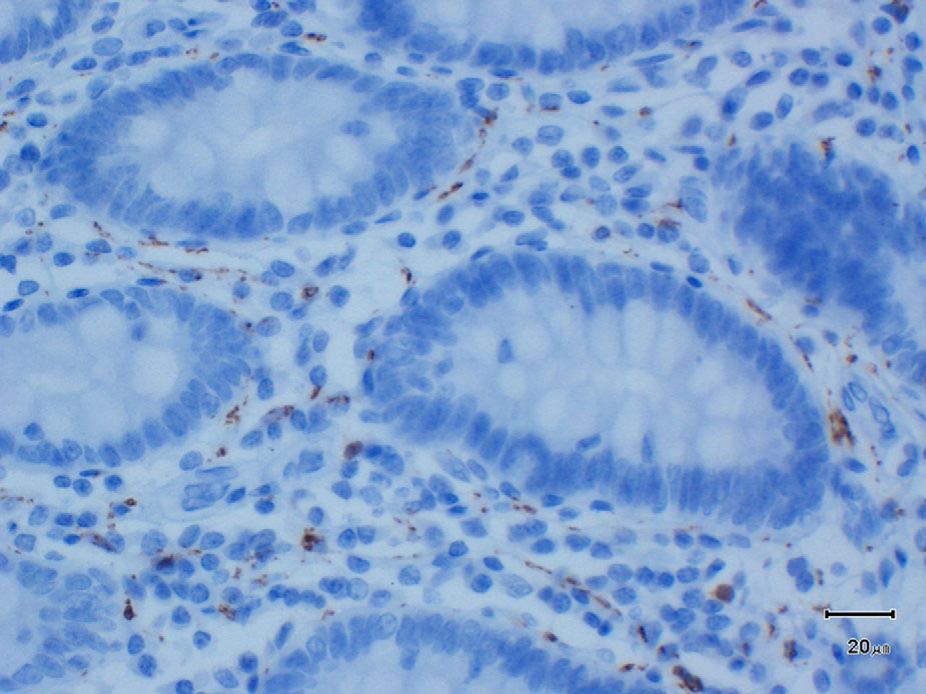
Staining of the colonic, mucosal nerve fiber network with calretinin (clone 5A5, 1:200 dilution). Magnification × 40. [Color figure can be viewed in the online issue, which is available at wileyonlinelibrary.com.]

Thus, there is clearly a need for more robust ways to distinguish neuronal ASN‐IRS from the nonneuronal staining frequently seen in the ENS. The type of ENS tissue available for the analysis should guide the choice of neuronal marker. For example, calretinin, a calcium‐binding protein used to diagnose Hirschsprung's disease (characterized by the absence of ganglionic cells) is particularly useful in detecting nerve fibers in the superficial layers of mucosa/submucosa (Fig. 2).^27^ On the other hand, in the whole‐wall samples containing the perikarya of myenteric and submucosal neurons, Abs specific for neuronal cell bodies (eg, anti‐Hu or PGP 9.5) can be more informative. Some studies have also compared the intensity of ASN staining between different neuronal subtypes by using Abs reactive for vasoactive intestinal polypeptide^17^ or tyrosine hydroxylase.^3^

Although DIF is useful to delineate the specificity of ASN staining into the neurons, morphological details are difficult to appreciate with this method. Of note, only 2 GI tract studies have shown both bright‐field images to highlight morphology and DIF to investigate the colocalization with nervous tissue, but even they applied different ASN‐Abs for each technique.^3^, ^5^ The majority of studies have used either one or the other technique, further hindering comparisons between studies.

In addition to aiding in localization of ASN staining, identification of nervous tissue can facilitate the investigation of neuronal loss in the GI tract in PD. Some studies have reported reduction in the density of myenteric ganglia in PD,^3^, ^28^ whereas others have not seen any neuronal loss.^29^

## Detection of Pathologic Species of ASN in the ENS

Clearly, how one defines the pathologic nature of ASN will have a direct impact on the sensitivity and specificity of ASN staining in the ENS. In PD, ASN aggregation in neurons is likely to begin in the distal synaptic axon terminals^30^ (where it most likely elicits its physiological function) and extend centripetally in the axons toward the neuronal soma, where LBs form.^31^ Interlaboratory comparisons have shown that different ASN Abs do not significantly differ in terms of LB counts but vary considerably in their ability to reveal ASN‐IRS in the neurites and axonal terminals.^32^, ^33^, ^34^ This antibody‐dependent variability in ASN expression is well known in the CNS^32^, ^33^, ^34^ but has also recently been demonstrated in the GI tract.^26^ Aldecoa and colleagues showed that some Abs detected diffuse, synaptic‐type staining reminiscent of physiological immunoreactivity, whereas others preferably labeled coarse ASN aggregates in the ganglionic soma and neurites that were considered pathological.^26^ Nonneuronal staining was also seen in the epithelial cells of gastric glands and vessel walls, in the smooth muscle cells of the muscularis propria, and in the leukocytes of the submucosa, as has been described by others.^13^ These studies highlight the limits of IHC in distinguishing between the pathological and physiological ASN staining patterns, both in neurons and in nonneuronal cells of the GI tract.^26^

To fully exploit the potential of colonic ASN as a biomarker, it is essential to understand the age‐ and site‐specific gene and protein expression patterns of ASN in the healthy ENS. Bottner and colleagues examined human rectosigmoid segments and observed the highest levels of ASN mRNA in the intestinal layers containing significant amounts of enteric neurons as well as in the laser microdissected ganglionic cells of healthy controls, regardless of age (ie, range, 15‐83 years).^13^ In a study on the submucosal plexus of healthy aging rats, Philips and coworkers found significant ASN expression throughout the animals' life span.^35^ Interestingly, they also reported increasing amounts of aggregated ASN in parallel with increasing age.^35^ These studies raise questions on the pathogenic nature of neuronal ASN and emphasize the need for further research investigating the biochemical nature of ASN in both neuronal and nonneuronal cells.

Posttranslational modifications of ASN such as phosphorylation (P‐ASN) have been suggested to define its transformation into a pathogenic molecule, because ∼90% of aggregated ASN within LBs extracted from the brain is phosphorylated at serine residue 129, compared with ∼4% phosphorylated ASN in the total brain.^36^ Thus, Abs reactive for phosphorylated ASN (P‐ASN‐Abs) have been used as a marker of pathologic ASN in the GI tract in most studies.^2^, ^3^, ^7^, ^13^, ^14^ Hilton and colleagues reported staining with P‐ASN‐Abs to be more extensive compared with that obtained with Abs against total ASN (T‐ASN‐Abs), although this was not formally quantified.^7^ Rather surprisingly, other studies found T‐ASN‐Abs to be more sensitive and specific in detecting PD patients than P‐ASN‐Abs.^8^, ^14^ One study described more intense staining with T‐ASN‐Abs compared with P‐ASN‐Abs in neurologically unimpaired subjects but also reported an age‐related increase in the density of the latter, suggesting that P‐ASN‐IRS may be age related.^13^ Furthermore, case‐control studies reporting 100% specificity for enteric ASN detection in PD do describe immunoreactivity with both T‐ and P‐ASN‐Abs in control cases but interpret this as “normal” or “nonspecific” staining.^5^, ^7^ Consistent graphic representation of these findings could aid the development of consensus on pathological and nonspecific staining patterns.

All in all, several studies have clearly demonstrated the presence of P‐ASN accumulation in the colon of healthy controls, which suggests that this may not be the “pathologic” form of the protein, but rather that ASN phosphorylation is a physiologically occurring process.^37^

There is also emerging evidence that the earlier intermediate species of the ASN aggregation process, that is, soluble, oligomeric aggregates of ASN, may ultimately be the pathogenic species underlying neuronal death in PD.^38^, ^39^ Thus, Abs reactive for oligomeric forms of ASN^40^ could improve specificity and sensitivity for pathological staining in the GI tract.

## Alternative Techniques

To date, conventional IHC has been the technique of choice to explore ASN pathology in the GI tract. By virtue of detecting also physiologic ASN, however, IHC has low specificity. There is thus a need for more sensitive and specific techniques to detect the accumulation of the earliest, pathological ASN species in the synaptic axonal terminals and neurites.

### Paraffin‐Embedded Tissue Blot

One such potential in situ technique is the paraffin‐embedded tissue (PET) blot, which was originally applied to detect the pathological prion protein in sporadic Creutzfeldt‐Jakob disease (sCJD).^41^ In PD and DLB brain, the ASN‐PET blot has revealed numerous synaptic ASN microaggregates in cortical and subcortical gray matter in addition to a relatively sparse number of LBs seen with conventional IHC.^30^ It is based on prolonged high‐concentration treatment with proteinase K (PK), an enzyme that digests any physiologic, monomeric ASN (enhancing specificity) but also reveals epitopes (increasing sensitivity).^42^ To date, 1 study has used this technique to detect synaptic ASN aggregates in the sigmoid colons and rectums of PD patients and neurologically unimpaired controls. With the ASN‐PET blot, sensitivity was 86% in the overall PD sample, whereas specificity was disappointingly dismal, with 100% of controls showing staining.^14^ The presence of PK‐resistant ASN in the PET blot of colonic tissue in all 11 neurologically unimpaired subjects certainly merits further investigation, as this technique was hypothesized to detect the pathological form of ASN alone. It is possible that medical conditions unrelated to PD could cause protein aggregation in the GI microenvironment. CNS tumors of various lineages have been linked to altered expression of 1 or more members of the synuclein family,^43^, ^44^ and γ‐synuclein is a well‐established marker of breast cancer.^45^ Considering that controls for this study were recruited from cancer screening programs, it is possible that mechanisms related to colonic neoplasms could have affected the state of aggregation of ASN in these subjects. Also, although fibrillar ASN from human Lewy body disease patients is more than 10 times more resistant to PK than its physiological, soluble counterpart, the PK treatment conditions applied by Visanji and coworkers may not have eliminated all the soluble ASN protein in the GI tract. Thus, experiments showing total elimination of physiological ASN with higher PK treatment or other denaturing conditions could shed more light on this method. Furthermore, although Visanji and coworkers used blocking peptides to demonstrate that their PET blot signal was specific for ASN, neuronal localization of the ASN staining was not immunohistochemically ascertained.^14^ Visanji and colleagues interestingly hypothesized that the reduction in the PK‐resistant ASN aggregation in PD compared with control colon could be a result of its disaggregation and release in the form of cytotoxic fibrillar oligomers, shown to occur in vitro.^46^ Thus, techniques that would allow detection of such ASN oligomers in situ in the colonic biopsies would be very useful.

### Proximity Ligation Assay

The proximity ligation assay (PLA) was originally developed for cancer research and has subsequently been used in cardiology, immunology, and microbiology.^47^, ^48^, ^49^ This technique is based on detection of simultaneous and proximal binding of target molecules (such as proteins) by 2 probes tagged with complementary strands of DNA. Consequent ligation and formation of circular DNA can then be amplified and easily visualized.^47^ In contrast to conventional IHC, PLA greatly increases sensitivity detecting concentrations of the target molecule in the zeptomolar range (10^‐21^ mol), whereas the required pairwise ligation increases specificity. The first PLA‐based assay to detect ASN oligomers in human PD brain tissue (ASN‐PLA) was recently introduced together with extensive data showing specificity of the assay with different in vitro ASN oligomerization systems.^50^ ASN‐PLA was shown to specifically label very early perikaryal aggregates (ie, pale bodies) but not late‐stage inclusions such as LBs. Moreover, AS‐PLA revealed previously unrecognized pathology in the form of extensive diffuse deposition of ASN, which was not detected by IHC or observed in controls.

### Protein Misfolding Cyclic Amplification and Real‐Time Quaking‐Induced Conversion Assays

None of the GI studies so far have examined the biochemical composition of ASN using standard Western blotting (WB), which is probably because of difficulty in obtaining in vivo frozen material from the ENS. However, WB would be more sensitive than IHC and would allow the quantitative assessment of pathological aggregated ASN. Availability of frozen tissue would also allow the application of other ultrasensitive techniques such as protein misfolding cyclic amplification (PMCA) and real‐time quaking‐induced conversion (RT‐QuIC) assays. Although both these techniques were originally developed to detect the pathological prion protein^51^, ^52^, ^53^ and serve as diagnostic tests for sCJD, they show the potential to be applied to other abnormally misfolded proteins such as ASN.^54^, ^55^ Importantly, rather than simply measuring the concentration of ASN, these techniques investigate the seeding capacity of ASN, that is, the ability of ASN to aggregate and induce further aggregation of nearby normal ASN. PMCA combines cycles of incubation at 37 °C (which promotes fibril formation) and sonication (which causes fragmentation of fibrils into smaller seeds), and the end product is detected by WB. Although PMCA provides faster kinetics of aggregation than RT‐QuIC, the sonication steps introduce variability hindering reproducibility. In RT‐QuIC, a recombinant protein is mixed or “seeded” with small amounts of aggregated protein from the patient's sample (eg, frozen GI biopsy). Interaction between recombinant and pathologic, patient‐derived misfolded protein is promoted by intermittent shaking (“quaking”), which leads to formation of further aggregates. These form bonds with thioflavin T generating a fluorescent signal that can be easily detected and quantified. It remains to be seen whether the assessment of functional properties of ASN on GI tissue will prove to have superior sensitivity and specificity for in vivo diagnosis of PD.

## ASN Detection in the ENS: Gateway to the Brain?

Already prior to the era of ASN IHC, characteristic inclusions histologically and ultrastructurally identical to LBs were found in various parts of the GI tract in patients with PD.^17^, ^56^, ^57^ However, this did not spark much scientific interest until the theory of prion‐like propagation was introduced in the field of PD research. According to this hypothesis, the accumulation of ASN in the dorsal motor nucleus of the vagus (DMV), generally considered to be the first vulnerable region of the CNS to become affected in PD, may actually originate in the ENS, where vagal fibers synapse with parasympathetic ganglionic neurons.^18^, ^58^ A putative pathogen capable of passing the mucosal lining of the GI tract might induce ASN misfolding in the terminal axons of postganglionic enteric neurons followed by retrograde transport to the cholinergic preganglionic neurons of the DMV.

Several studies report how intracerebral injection of exogenous, preformed fibrillar ASN in the brain of various mouse models of PD induces a progressive ASN‐IRS pattern that suggests propagation of ASN pathology via a prion‐like, conformational self‐templating mechanism.^59^, ^60^ Experimental evidence for the spread of ASN pathology from the PNS to the CNS via peripheral nerves comes from studies in which preformed amyloidogenic ASN filaments were injected into the intestinal wall or the hind leg muscle of transgenic and wild‐type mice.^61^, ^62^ ASN was transported via the vagus nerve and reached the DMV in a time‐dependent manner.^62^ Mice with intramuscular injection also developed a relatively rapid, widespread induction of ASN inclusion pathology in the CNS, which was significantly delayed or even prevented by sciatic nerve transection.^61^ However, the soluble nonamyloidogenic form of ASN (Δ71‐82) also induced CNS ASN‐IR inclusions, suggesting that other mechanisms apart from conformational self‐templating could be involved.^61^ Further mechanistic insight comes from animal models showing that chronic oral ingestion of low‐dose rotenone can lead to accumulation of ASN in the ENS ganglia. This was followed by ASN accumulation in the DMV and SN, progress that was prevented by vagotomy.^63^, ^64^

Although the vagal connection from the ENS to the DMV is often invoked to account for the transmission of ASN pathology, it should be noted that a number of studies have examined the distal GI tract, more readily accessible to routine biopsies (Fig. 1).^5^, ^14^ This region, however, is not reached by vagal projections according to previous anatomical studies in humans and animals.^65^, ^66^ There is a general consensus on the distribution of vagal innervation to the GI tract, which falls progressively following a rostrocaudal gradient: 50% of ENS ganglia receive vagal input at the level of the ascending colon, whereas there is no vagal input in the descending colon or rectum, which receive parasympathetic innervation from the sacral plexus and pelvic nerves. Thus, propagation of misfolded ASN from the distal colon and rectum would potentially require an extra passage in the rostral direction, possibly through enteric interneurons, to reach the vagal nerve endings. Alternatively, misfolded ASN in the distal colon and rectum could travel retrogradely to the preganglionic parasympathetic neurons in the sacral plexus. However, according to anatomical studies by Braak and colleagues,^67^ this region is not involved by ASN pathology until later stages of disease and would thus not seem to be linked to early ASN accumulation in the distal GI tract. Furthermore, the neurons that do receive vagal fibers are almost exclusively found in the myenteric plexus,^65^ whereas reported staining in PD is most often confined to mucosal nerve fibers or to the submucosal ganglionic cells where the fibers originate. Thus, one would need to account for this further step in the PNS‐CNS pathway linking the mucosal nerve fibers emanating from submucosal neurons to the myenteric plexus.^68^

To validate whether the peripheral ASN pathology precedes or occurs concomitantly to that occurring in the CNS and whether it progresses in a predictable way as has been proposed,^69^ we need further confirmatory, human postmortem studies assessing both the brain and the ENS. Although there are case reports on ASN‐IRS in the PNS without concomitant Lewy pathology in the CNS,^70^ the majority of postmortem studies investigating both CNS and PNS fail to provide evidence of centripetal spread of ASN.^10^, ^18^, ^19^, ^71^ Taken together, these findings suggest that CNS ASN deposition may in fact precede PNS involvement. Interestingly, to our knowledge no experimental models have yet investigated the spread of ASN pathology in the other direction (ie, CNS to PNS), as has been reported to occur with prion transmission.^72^ It is also plausible that ASN deposition in the PNS could serve as a focal biomarker, not necessarily spreading anywhere but occurring simultaneously with the brain pathology and thus serving as a surrogate marker.

## What Kind of Biomarker Could Enteric ASN Be?

A prerequisite for the development of preventive treatment for PD is the ability to diagnose the disease before a significant loss of nigral neurons has occurred. A recent consensus paper by the Movement Disorder Society task force emphasized the need to define “preclinical” and “prodromal” PD and develop biomarkers for PD.^73^ Assuming it will become possible to reliably define the presence of pathological ASN in the ENS that distinguishes patients with PD from controls, such a biomarker could have several potential applications.

Detection of ASN‐IRS in colonic biopsies taken up to 8 years prior to the onset of PD motor symptoms supports the potential of GI ASN as a presymptomatic diagnostic biomarker. To date, 6 subjects with presymptomatic GI ASN‐IRS have been described in 2 separate studies.^7^, ^11^ Of note, review of medical charts in 3 of these individuals failed to reveal constipation in the premotor phases. To understand how early in the disease process we see the enteric ASN accumulation and how it correlates with the disease progression, systematic screening should be performed on asymptomatic subjects with established risk factors for PD (eg, REM sleep behavior disease order and genetic mutation carriers). Results from longitudinally followed, uniformly assessed cohorts could more reliably confirm or refute enteric ASN detection as a predictive marker and its role in premotor gastrointestinal impairment.

Another possibility is that ASN may be consistently detectable only after the onset of motor symptoms, in which case it could be used to confirm the diagnosis made on clinical grounds. To increase specificity in this context, further studies are needed assessing GI ASN staining in other, “atypical” parkinsonian syndromes. Although the postganglionic neurons are generally spared in multiple system atrophy, 1 study reported colonic ASN‐IRS in 1 of 6 patients.^74^ Progressive supranuclear palsy is a tauopathy and would thus not be expected to disclose significant ASN pathology.

To date, only 1 study has attempted clinicopathological correlation, reporting an association between more severe colonic ASN staining and levodopa‐unresponsive symptoms such as dysarthria and postural instability.^3^ Thus, phenotypic stratification in relation to enteric ASN accumulation is a rather unexplored area but is expected to evolve once more standardized criteria for the definition and quantification of pathology are established.

Finally, ASN staining in the GI tract may characterize only a proportion of cases of “idiopathic” PD. For example, one could hypothesize that a certain percentage of individuals will be affected by ASN pathology originating from the nasal mucosa, whereas in others the possible spread of pathology might commence in the ENS.

## Conclusions and Future Work

We have discussed the several sources of heterogeneity that need to be addressed prior to validation of GI ASN detection as a tissue‐based diagnostic tool for PD. Ideally, these should be addressed in the context of a cooperative, multicenter effort to establish consensus on the best method to use for a robust and informative ASN detection in the GI tract (Table 2). Although there is consistent evidence of different species of ASN, currently available techniques cannot distinguish reliably between physiological and pathological forms of ASN. Alternative detection methods such as PET blot, PLA, PMCA, and RT‐QuIC may prove to be more sensitive and specific tools for the detection of early, disease‐associated ASN aggregation in the axonal terminals and neurites of the GI tract, where the pathological process most likely starts. Future studies should also apply software‐based image analysis for a more accurate and reproducible quantification of pathological changes. For the detection of GI ASN to be clinically useful, these studies should be based on longitudinally assessed, population‐derived case‐control cohorts with extensive clinical characterization.

**Table 2:** Issues hindering consensus on the optimal method for reliable and reproducible GI ASN detection

Source of heterogeneity	Suggested consensus approach
ASN‐reactive antibody	Use at least 2 antibodies reactive for different epitopes and/or variants of ASN (eg, P‐ASN and T‐ASN), on the same number of consecutive sections. Consider antibodies reactive for oligomeric ASN.
Biopsy site	For use as clinical biomarker, prioritize low discomfort for patient (ie, flexible sigmoidoscopy) and reproducibility. For research into pathogenic mechanisms, prioritize vagal innervation (ie, esophagus and stomach).
Amount of stained tissue	Apply software‐based image analysis (SBIA) to adjust for variability in stained area between different biopsy samples.
Definition of pathological staining	Increase sharing of images. Apply reproducible SBIA algorithms. Be aware of nonneuronal ASN staining patterns.
Neuronal marker	Always use at least 1 reliable marker of nervous tissue. Selection should take into account characteristics of available tissue (superficial versus whole wall).
